# Bilateral Habenula deep brain stimulation for treatment-resistant depression: clinical findings and electrophysiological features

**DOI:** 10.1038/s41398-022-01818-z

**Published:** 2022-02-03

**Authors:** Chencheng Zhang, Yingying Zhang, Huichun Luo, Xinmeng Xu, Ti-fei Yuan, Dianyou Li, Yi-yun Cai, Hengfen Gong, Dai-hui Peng, Yi-ru Fang, Valerie Voon, Bomin Sun

**Affiliations:** 1 Department of Neurosurgery, Center for Functional Neurosurgery, Clinical Neuroscience Center, Ruijin Hospital, Shanghai Jiao Tong University School of Medicine, Shanghai, China; 2 Shanghai Center for Brain Science and Brain-inspired Technology, Shanghai, China; 3 Shanghai Key Laboratory of Psychotic Disorders, Shanghai Mental Health Center, Shanghai Jiao Tong University School of Medicine, Shanghai, China; 4 Clinical Research Center and Division of Mood Disorders, Shanghai Mental Health Center, Shanghai Jiao Tong University School of Medicine, Shanghai, China; 5 Department of Psychiatry, Pudong District Mental Health Center, Shanghai, China; 6 Department of Psychiatry, University of Cambridge, Cambridge, United Kingdom

**Keywords:** Diseases, Depression

## Abstract

Deep brain stimulation (DBS) of structures in the brain’s reward system is a promising therapeutic option for patients with treatment-resistant depression (TRD). Recently, DBS of the habenula (HB) in the brain’s anti-reward system has also been reported to alleviate depressive symptoms in patients with TRD or bipolar disorder (BD). In this pilot open-label prospective study, we explored the safety and clinical effectiveness of HB–DBS treatment in seven patients with TRD or BD. Also, local field potentials (LFPs) were recorded from the patients’ left and right HB to explore the power and asymmetry of oscillatory activities as putative biomarkers of the underlying disease state. At 1-month follow-up (FU), depression and anxiety symptoms were both reduced by 49% (*n* = 7) along with substantial improvements in patients’ health status, functional impairment, and quality of life. Although the dropout rate was high and large variability in clinical response existed, clinical improvements were generally maintained throughout the study [56%, 46%, and 64% reduction for depression and 61%, 48%, and 70% reduction for anxiety at 3-month FU (*n* = 5), 6-month FU (*n* = 5), and 12-month FU (*n* = 3), respectively]. After HB–DBS surgery, sustained improvements in mania symptoms were found in two patients who presented with mild hypomania at baseline. Another patient, however, experienced an acute manic episode 2 months after surgery that required hospitalization. Additionally, weaker and more symmetrical HB LFP oscillatory activities were associated with more severe depression and anxiety symptoms at baseline, in keeping with the hypothesis that HB dysfunction contributes to MDD pathophysiology. These preliminary findings indicate that HB–DBS may offer a valuable treatment option for depressive symptoms in patients who suffer from TRD or BD. Larger and well-controlled studies are warranted to examine the safety and efficacy of HB–DBS for treatment-refractory mood disorders in a more rigorous fashion.

## Introduction

Major depressive disorder (MDD) is a common psychiatric disorder with an estimated lifetime prevalence of 14.6% in high-income countries [1]. Effective therapeutic options for MDD include psychotherapy, multiple classes of antidepressants, and electroconvulsive therapy (ECT). Nevertheless, as many as 30% of patients do not respond to multiple consecutive antidepressant strategies [2], while 52% of medication-resistant patients do not respond to ECT [3]. Such patients, who are often referred to as having treatment-resistant depression (TRD), may be considered to be in the advanced stage of depression [4]. Compared with non-TRD patients, they have a higher hospitalization rate, more suicide attempts, and higher treatment costs. Patients with TRD are also characterized by severe functional impairment, reduced work productivity, high disease burden, and clear unmet treatment needs [5–7].

Deep-brain stimulation (DBS) is a promising therapeutic option for patients who suffer from TRD, conceivably by modulating pathological activity of the brain regions and networks that are involved in the illness [8]. DBS involves implanting electrodes into certain brain regions, delivering electric impulses through the electrodes, and optimizing stimulation parameters (e.g., voltage, frequency, and pulse width). Since 2005, several open-label trials have reported promising clinical effects of DBS in TRD, mainly targeting brain structures implicated in the neurobiology of MDD and the brain’s “reward” system, which mediates positive motivations that drive people to seek out pleasure and rewarding stimulus events. The main DBS targets examined in these studies include the subcallosal cingulate gyrus (SCG), medial forebrain bundle (MFB), ventral capsule/ventral striatum (VC/VS), and nucleus accumbens (NAc). Reported response rates, defined as a symptom decrease of at least 50%, range from 30% to 90%, with most studies reporting a response rate of around 50% [9]. Furthermore, the antidepressant effects of DBS may be maintained across the long-term course of illness [10]. The SCG and VC/VS have been the two most widely used targets, but SCG can take a few years to achieve its therapeutic effects, while DBS of VC/VS carries risk of inducing hypomania [8, 10]. These clinical findings are encouraging, although two early large randomized, sham-controlled trials, one targeting the SCG [11] and the other the VC/VS [12], failed to demonstrate efficacy of DBS treatment for TRD.

In recent years, there has been a growing interest in the role of the habenula (HB) in the brain’s anti-reward system and its potential role in the pathophysiology of mood disorders [13], especially MDD and bipolar disorder (BD). In contrast to the reward system, the anti-reward system mediates negative motivations, thereby inhibiting behaviors in response to unpleasurable, aversive events, such as pain, stress, and fear-provoking stimuli. Moreover, the HB plays a crucial role in the brain mechanisms of sleep and wakefulness. Given its role in behavioral activation and negative motivational states, dysfunction of the HB may also play a significant pathophysiological role in mood disorders, in addition to structures located in the brain’s reward system. In line with this hypothesis, experimental ablation of the HB in animal models of MDD alleviates depressive-like behavior [14]. Furthermore, a clinical study found that the severity of depression symptoms in a patient with TRD was substantially reduced after DBS targeting the major afferent bundle (stria medullaris) of the lateral HB [15]. Moreover, we reported a significant improvement in depression in a patient with medically intractable BD after DBS of the HB [16]. In the latter study, the patient’s manic symptoms were not affected by HB–DBS, which supports the view that the HB contributes to negative motivational states instead of positive motivational states.

Further support for this hypothesis comes from a recent study in which we recorded local field potentials (LFPs) from HB–DBS electrodes in patients with psychotic or mood disorders while they were viewing pictures with positive, negative, and neutral emotional valence [17]. Patients showed a distinct power increase of HB LFP activities in the theta/alpha (5–10 Hz) band upon the presentation of the emotionally negative stimuli. Moreover, as assessed by simultaneous recording of magnetoencephalography, the emotionally negative pictures were associated with increased theta/alpha activity in the prefrontal cortex, as well as with increased theta/alpha synchronization between HB and prefrontal cortex. These data support the view that the HB and its interaction with the prefrontal cortex may have a particularly important role to play in encoding emotionally negative stimulus events [17].

In sum, preclinical and clinical studies suggest that HB–DBS could be a valuable alternative treatment option for patients who suffer from TRD. Furthermore, HB–DBS could be useful in the management of depressive episodes in patients with BD, without carrying risk for inducing hypomania. Therefore, we evaluated the clinical effectiveness and adverse-effect profile of HB–DBS treatment in patients with TRD and BD. Moreover, we explored the relationships between the severity of the patients’ clinical symptoms at baseline and features of LFPs recorded from the patients’ left and right HB. We hypothesized that symptom severity would be associated with the power of HB LFP oscillatory activity, assuming that this spectral feature accurately reflects the role of HB in negative motivational states [17]. Also, based on EEG and MEG studies implicating brain asymmetry in affective function and dysfunction [17], we hypothesized that symptom severity would be associated with the extent of synchronization and lateralization of LFP oscillatory activities between the left and right HB.

## Materials and method

### Participants

This study included a series of patients who took part in a HB–DBS clinical trial for TRD. They fulfilled the following trial inclusion criteria: (1) presence of a current mood disorder, involving a primary clinical DSM-5 diagnosis of either MDD or BD, with a chronic illness episode for over 2 years or a recurrent illness with at least four lifetime episodes; (2) presence of moderate-to-severe depression symptoms, as defined by having a score of 18 or higher on the 17-item Hamilton Depression Rating Scale (HAMD); (3) illness duration, defined as the time from the first depressive episode to HB–DBS surgery, of at least 5 years; (4) history of failure to clinically respond to multiple classes of medication (at least two types of medications for MDD and at least three types for BD) or to at least one session of electroconvulsive therapy; and (5) not taking medication or on stable medication regimen for at least one month before study entry. Exclusion criteria included (1) a current or past psychotic disorder, (2) the presence of a personality disorder, and (3) any medical contraindication to surgery. Medication titration was not allowed during the study. All participants provided written informed consent. The study was approved by the Ethics Committee of Ruijin Hospital, Shanghai Jiao Tong University School of Medicine (No. 2017-172) and registered at www.clinicaltrial.gov (NCT03347487).

### Surgical procedure

High-resolution magnetic resonance imaging (MRI, 3.0 T, General Electric Company, USA) and Leksell SurgiPlan (Elekta, Stockholm, Sweden) were used for targeting the HB in each patient’s brain. From each patient, we obtained T1 maps, proton spin-density maps, R2* maps, and quantitative susceptibility maps. Using the patient’s high-resolution T1-weighted MRI images, we were able to delineate the HB clearly in the axial view. The target site was located about 0–2 mm in front of the posterior commissure, 3–5 mm lateral, and 0–1 mm above the anterior commissure–posterior commissure.

Both quadripolar DBS electrodes (model 3389, Medtronic, Minneapolis, MN, USA; or 1210-40, SceneRay, Suzhou, China) and the pulse generator (37603 SC, Medtronic; or 1180, SceneRay) were implanted under general anesthesia. DBS electrode contacts had a length of 1.5 mm and a diameter of 1.27 mm, and the spacing between contacts was 0.5 mm. DBS parameters used for each patient are listed in Supplementary Table 1. The DBS programming procedure we followed has been described by Bergfeld et al. [8].

### Clinical outcome assessment

Clinical assessments were conducted before HB–DBS surgery (baseline) and at 1-, 3-, 6-, and 12-month follow-up (FU). The following clinical outcomes were assessed: (1) severity of depression symptoms, as assessed by the HAMD; patients were classified as responders if their HAMD scores decreased by ≥50%, partial responders if their HAMD scores decreased by ≥25%, and nonresponders if their HAMD scores decreased by <25%; (2) severity of mania symptoms as evaluated by the Youth Mania Rating Scale (YMRS); (3) severity of anxiety symptoms as evaluated by means of the 14-item Hamilton Anxiety Rating Scale (HAMA); (4) sleep quality as measured by the Pittsburgh Sleep Quality Index (PSQI, with higher scores indicting lower sleep quality); and (5) health status, functional impairment (in work/school, social, and family life), and quality of life (QoL) as assessed by the Medical Outcome Studies (MOS) 36-item Short Form Health Survey (SF-36), the Sheehan Disability Scale (SDS), and the World Health Organization (WHO) Quality of Life Questionnaire (WHOQOL-BREF).

### Electrophysiological data recording

Within 2–5 days after DBS electrode implantation, intracerebral LFPs were recorded while the patient was at rest and seated. The recordings were performed before turning on the HB–DBS. For each patient, 5-minute resting unipolar LFPs were recorded from four DBS electrode contacts, with a common electrode placed on the surface of the mastoid. The LFPs were recorded using a BrainAmp MR amplifier (Brain Products GmbH, Gilching, Germany) with a sampling rate of 1000 Hz. For each electrode, three-channel bipolar LFPs involving four unipolar recordings were obtained. Here, we focus on the LFPs recorded from the HB contacts that were used for postoperative therapeutic optimization and continuous stimulation.

The location of the electrodes was reconstructed based on preoperative MRI and postoperative CT. The calculation and 3D visualization (Supplementary Fig. 1) were performed by the Lead-DBS toolbox (Horn et al., 2015, [3]). LFP recordings were obtained from 6 out of 7 patients who enrolled in the study because one patient preferred not to participate in the electrophysiological examination. Overall, a total of 12 LFP recordings (6 recordings from the left HB electrode and 6 from the right HB electrode) were used for analysis of LFP oscillatory activities.

### Electrophysiological data analysis

The analysis of the LFP data was performed offline with custom-developed scripts in MATLAB (MathWorks Inc., Natick, MA, USA). LFP data preprocessing steps were (a) low-pass filtering at 90 Hz with a Chebyshev Type I filter to rule out LFP oscillatory activities not of interest, (b) high-pass filtering at 2 Hz with a Chebyshev Type I filter to eliminate baseline shifting, (c) applying an adaptive notch filter to remove 50 Hz line noise, (d) downsampling to 500 Hz, and (e) segmenting into 2-sec epochs, excluding epochs (trials) containing signal-voltage amplitudes exceeding six times the average value. Subsequently, the preprocessed segmented LFP data between 2 and 90 Hz were decomposed into 10 different frequency-band oscillations: theta (*θ*, 4–7 Hz), alpha (*α*, 8–12 Hz), low-beta (l*β*, 13–20 Hz), high-beta (h*β*, 21–30 Hz), gamma1 (*γ*1, 31–40 Hz), gamma2 (*γ*2, 41–50 Hz), gamma3 (*γ*3, 51–60 Hz), gamma4 (*γ*4, 61–70 Hz), gamma5 (*γ*5, 71–80 Hz), and gamma6 (*γ*6, 81–90 Hz).

Two features of the extracted LFP frequency-band oscillations were quantified: (1) power spectral density (PSD), which evaluates the strength of the oscillations within each frequency band; and (2) an asymmetrical index (AI), which evaluates asymmetrical oscillatory activity between the left and right HB.

First, the PSD was calculated using a fast Fourier transform with a 2-sec sliding window and a 1-sec overlap. To reduce the influence of interindividual variability, the power spectra were normalized by dividing the values by the integral power between 2 and 90 Hz. Finally, for each patient, the normalized PSD values of all trials were averaged.

Second, the AI of the HB with a specific frequency-band oscillation was computed as the difference between the natural–logarithmetic transformed scores of the left and right oscillation powers:

1 $${{{\mathrm{AI}}}} = {{{\mathrm{ln}}}}\left( {{{{\mathrm{P}}}}_{{{\mathrm{L}}}}} \right) - {{{\mathrm{ln}}}}\left( {{{{\mathrm{P}}}}_{{{\mathrm{R}}}}} \right)$$

The P_L_ and P_R_ represent the power values of the same frequency-band oscillation recorded from the left and right HB, respectively. If the AI value is higher than 0, the oscillatory activity from the left HB is stronger than that from the right HB. Conversely, if the AI is lower than 0, the oscillatory activity from the left HB is weaker than that from the right HB. If AI = 0, the oscillatory activities between the left and right HB are fully balanced.

### Statistical analysis

We used paired-sample t-tests to compare the patients’ scores on the HAMD, HAMA, PSQI, SF-36, SDS, and WHOQOL-BREF obtained at baseline with their corresponding scores obtained at 1-, 3-, 6-, and 12-month FU. We used the Spearman’s rank-order correlation to assess the associations between the quantified LFP data (PSD and AI) and the clinical data. The critical level of significance for all tests was set at 0.05 (two-tailed). Additionally, permutation testing was performed to evaluate the statistical significance of the quantified LFP data. The threshold *P*-value was 0.05 and the permutation number was 1000. All reported P-values are based on permutation-significance testing. Because substantial interindividual differences in clinical response were anticipated, we also present individual patient data along with the group-averaged data and corresponding statistical results. Data values presented in the text represent mean and standard deviation, unless otherwise indicated.

## Results

### Patient characteristics

Table 1 presents the demographic and clinical characteristics of each of the seven patients (five males and two females, age: 39.0 ± 8 years) enrolled in this pilot study. Six patients were diagnosed with BD (4 with BD-I and 2 with BD-II) and one patient with MDD using the DSM-5 criteria. The patients’ age of illness onset was 26 ± 9 years. None of the patients had a family history of MDD. Diagnosed psychiatric comorbidities were present in 3 out of the 7 patients, with two patients presenting with a comorbid generalized-anxiety disorder (GAD) and one patient with a comorbid substance-use disorder (SUD).

**Table 1:** Demographic and clinical data for each patient included in study.

	Patient 1	Patient 2	Patient 3	Patient 4	Patient 5	Patient 6	Patient 7	Group
Gender	M	F	M	F	M	M	M	2 F/5 M
Marriage status	Married	Single	Married	Single	Married	Divorced	Divorced	2 single/3 married/ 2 divorced
Education (yr)	12	12	13	18	16	16	9	14 ± 3
Current age (yr)	41	30	48	28	35	48	45	39 ± 8
Age at MDD onset	20	25	39	18	16	36	36	27 ± 9
No. lifetime episode	7	1	7	1	5	4	1	4 ± 2
Current episode(yr)	3.5	5	1	10	19	1	10	7 ± 6
DSM-5 Diagnosis	BDI	BDI	BDI	BDII	MDD	BDII	BDI	4 BDI/2 BDII/1 MDD
Comorbidity	no	no	no	no	GAD	GAD	SUD	3 of 7
Past ECT	yes	no	yes	yes	no	yes	yes	5 of 7
Past TMS	no	no	yes	yes	yes	yes	no	4 of 7
Past psychotherapy	no	yes	no	yes	no	yes	no	3 of 7
Family history MDD	no	no	no	no	no	no	no	0 of 7

*MDD* major depressive disorder, *GAD* generalized anxiety disorder, *SUD* substance use disorder, *ECT* electroconvulsive therapy, *TMS* transcranial magnetic stimulation, *DSM-5* diagnostic and statistical manual of mental disorders, Version 5; *BDI* bipolar disorder Type I, *BDII* bipolar disorder type II.

### Clinical data

**Fig. 1:**
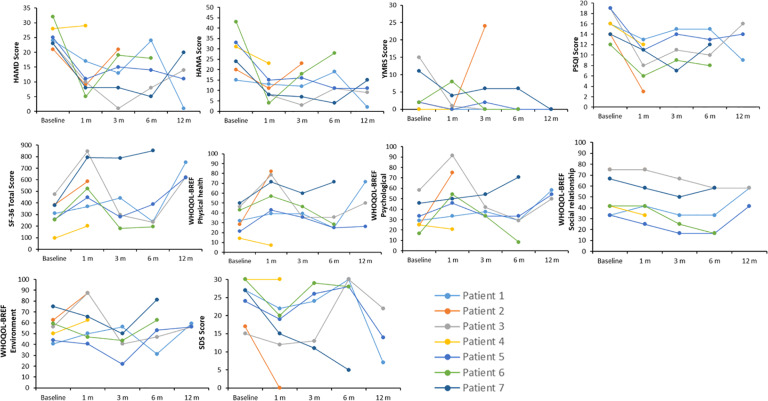
Clinical outcomes. HAMD 17-item Hamilton Depression Rating Scale, HAMA 14-item Hamilton Anxiety Rating Scale, YMRS Young Mania Rating Scale, PSQI Pittsburgh Sleep Quality Index, WHOQOL-BREF World Health Organization Quality of Life-BREF, SF-36 MOS Short Form Health Survey, SDS Sheehan Disability Scale, 1 m 1 month after surgery, 3 m 3 months after surgery, 6 m 6 months after surgery, 12 m 12 months after surgery.

The individual patient data obtained at baseline and each FU are illustrated in Fig. 1. Because four patients dropped out before completing all FU assessments, we first describe these patients and the reasons for withdrawing from the study before reporting the clinical outcome and LFP data.

#### Patient dropout

Patients 2 and 4 were lost to 3-month FU. Patient 2 was a 30-year-old female who suffered from BD for about 5 years at study entry, but she experienced an acute manic episode about 2 months after surgery. Although she showed a good clinical response to HB–DBS at 1-month FU (e.g., her HAMD and YMRS scores changed from 21 and 2 at baseline to 9 and 0 at 1-month FU, respectively), the patient’s clinical state subsequently worsened (HAMD score of 21 and YMRS score of 24 at 3-month FU) to such an extent that hospitalization was required. Patient 4 was a 28-year-old female who had lived with BD for about 10 years at study entry. She was classified as a nonresponder at 1-month FU and experienced uncomfortable sensations and feelings when the DBS was turned on, in addition to experiencing other adverse side effects, as discussed later. At 1-month FU, her physical health status also declined (e.g., 50% reduction in WHOQOL-BREF physical health score). Ultimately, she questioned the usefulness of the DBS for her symptoms and requested to discontinue the stimulation 2 months after surgery.

Patients 6 and 7 were lost to 12-month FU. Patient 6 was a 48-year-old male diagnosed with a comorbid GAD who had been ill for about 8 years at study enrollment. After surgery, his depression and anxiety symptoms improved, though his mania symptoms increased, at 1-month FU. His mania symptoms resolved thereafter, but the postsurgical improvement in depression and anxiety was only partially maintained at 3- and 6-month FU. He became less engaged over the DBS treatment course and experienced a significant decline in physical and mental health. He lost interest in the DBS intervention, requested other therapy, and withdrew from our study. Patient 7 was a 45-year-old male diagnosed with a comorbid SUD who had been ill for about 9 years at study entry. He showed a good clinical response to HB–DBS at 1-, 3-, and 6-month FU, but his depression and anxiety symptoms (though not his mania symptoms) recurred after he had stopped taking his antidepressant medication about 11 months after surgery. Due to his failure to maintain medication adherence, the patient’s clinical data collected at 12-month FU were excluded from the analysis.

Thus, the analysis of the clinical outcome data at 1-, 3-, 6-, and 12-month FU included data from 7 patients, 5 patients, 5 patients, and 3 patients, respectively.

#### Depression symptoms

Relative to baseline, the severity of depression symptoms in the seven patients enrolled was reduced by about 49% at 1-month FU (HAMD score: 25.1 ± 3.7 vs. 12.7 ± 8.1; *t* (6) = 3.9, *p* = 0.008). The clinical improvement observed at 1-month FU was maintained at 3-month FU (56% symptom reduction, HAMD score: 25.4 ± 3.8 vs. 11.2 ± 6.9; *t* (4) = 6.7, *p* = 0.003), 6-month FU (46% reduction, HAMD score: 25.4 ± 3.8 vs. 13.8 ± 7.6; *t* (4) = 3.7, *p* = 0.020), and 12-month FU (64% reduction, HAMD score: 24.0 ± 1.0 vs. 8.7 ± 6.8; *t* (2) = 3.7, *p* = 0.065). Clinical response rates, including full and partial responders, were 86%, 80%, 80%, and 100% at 1-, 3-, 6-, and 12-month FU, respectively. Among the 3 patients classified as responders at 12-month FU, two patients (Nos. 3 and 5) were similarly classified as partial or full responders at the prior FU assessments. The remaining patient (No. 1) responded less consistently across the treatment course, being classified as partial responder at 1- and 3-month FU, nonresponder at 6-month FU, and full responder at 12-month FU (Fig. 1).

#### Anxiety symptoms

The severity of anxiety symptoms was reduced by about 49% at 1-month FU (HAMA score: 27.1 ± 9.3 vs. 11.7 ± 6.2; *t* (6) = 3.5, *p* = 0.014), 61% at 3-month FU (HAMA score: 27.8 ± 10.6 vs. 11.2 ± 6.2; *t* (4) = 4.5, *p* = 0.011), 48% at 6-month FU (HAMA score: 27.8 ± 10.6 vs. 14.6 ± 9.2; *t* (4) = 2.9, *p* = 0.045), and 70% at 12-month FU (HAMA score: 24.0 ± 9.0 vs. 7.3 ± 4.7; *t* (2) = 6.1, *p* = 0.026). At the individual level, all patients had a lower HAMA score at their final FU than that at baseline (Supplementary Fig. 1), with score reductions ranging from 26% to 87%. Anxiety-symptom-score reductions for the two patients with comorbid GAD were 67% (patient 5 at 12-month FU) and 35% (patient 6 at 6-month FU).

#### Mania symptoms

At the group level, the patients’ symptoms of mania were substantially reduced following HB–DBS surgery, but the observed symptom reductions did not reach the level of statistical significance. Specifically, mania symptoms were reduced by about 59% at 1-month FU (YMRS score: 4.6 ± 5.9 vs. 1.9 ± 3.1; *t* (6) = 1.1, *p* = 0.297), 73% at 3-month FU (YMRS score: 6.0 ± 6.6 vs. 1.6 ± 2.6; *t* (4) = 1.6, *p* = 0.192), 80% at 6-month FU (YMRS score: 6.0 ± 6.6 vs. 1.2 ± 2.7; *t* (4) = 1.8, *p* = 0.147), and 100% at 12-month FU (YMRS score: 5.7 ± 8.2 vs. 0.0 ± 0.0; *t* (2) = 1.2, *p* = 0.351).

Again, large interindividual differences in clinical response were present, with patients 1 and 4 manifesting no symptom reductions (YMRS scores of 0 at baseline and final FU), patient 7 showing substantial (46%) reduction (YMRS score changed from 11 to 6), and patients 3 and 5 achieving large (100%) symptom reductions (YMRS scores changed from 15 to 0 and 2 to 0, respectively). Patient 6 initially displayed an increase in mania symptoms at 1-month FU (YMRS score changed from 2 to 8), but his symptoms were resolved thereafter (YMRS score of 0 at both 3- and 6-month FU). Patient 2, who was lost to 3-month FU, exhibited reduced mania symptoms at 1-month FU (YMRS score changed from 2 to 0) before experiencing an acute manic episode shortly thereafter.

#### Sleep quality

The patients’ sleep quality showed significant and sustained improvements after HB–DBS surgery, reaching up to a 42% improvement at 1-month FU [PSQI mean-score difference: *t*(6) = 5.0, *p* = 0.003], 30% at 3-month FU [*t*(4) = 3.8, *p* = 0.020], 28% at 6-month FU [*t*(4) = 3.1, *p* = 0.037], and 28% at 12-month FU [*t*(2) = 4.3, *p* = 0.049]. At the individual level, all patients had lower PSQI scores at their final FU than at baseline.

#### Health status, functional impairment, and quality of life

The patients’ health status, functional impairments, and QoL exhibited moderate-to-large improvements that were generally more distinct at 1- and 12-month FU than at 3- and 6-month FU. Specifically, the patients’ health status was improved by 75% at 1-month FU [SF-36 score difference, *t*(6) = 4.6, *p* = 0.004] and 91% at 12-month FU [*t*(2) = 3.6, *p* = 0.069], but it was improved only by 19% at 3-month FU [*t*(4) = 0.6, *p* = 0.569] and 14% at 6-month FU [*t*(4) = 0.8, *p* = 0.721]. Similarly, the patients’ functional impairments in school/work, social, and family life were reduced by 31% at 1-month FU [SDS mean-score difference: *t*(6) = 3.4, *p* = 0.015] and 35% at 12-month FU [*t*(2) = 1.0, *p* = 0.433]. Their functioning was improved by 16% at 3-month FU [*t*(4) = 1.3, *p* = 0.268], while showing no change at 6-month FU [*t*(4) = 0.1, *p* = 0.951]. Additionally, the patients’ overall quality of life was improved by 61% at 1-month FU [WHOQOL-BREF mean-score differences: *t*(6) = 2.8, *p* = 0.030] and 49% at 12-month FU [*t*(2) = 1.4, *p* = 0.299], with only a slight (13%) improvement at 3-month FU [*t*(4) = 1.2, *p* = 0.303] and a near-zero percentage improvement at 6-month FU [*t*(4) = 0.2, *p* = 0.858].

At the individual level, all patients (except patient 6) showed a higher-than-baseline SF-36 score and a higher overall WHOQOL-BREF score at the final FU. Also, all patients manifested a lower-than-baseline SDS score at the final FU, except for patient 4 who obtained the same score and patient 3 who exhibited a higher score, indicative of increased functional impairment.

### Electrophysiological data

**Fig. 2:**
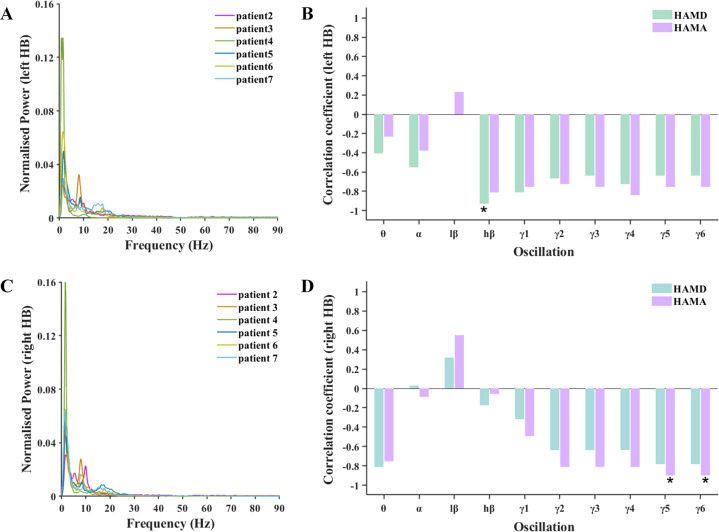
Electrophysiological results of power spectral density. The normalized power-spectral density (PSD) of LFP oscillations from left HB (Panel **A**) and right HB (Panel **C**) and their correlations with baseline HAMD and HAMA scores (Panels **B** and **D**). Patient 1 is not indicated because he declined to participate in the LFP recording session. In Panels **B** and **D**, correlations reaching statistical significance are indicated by *(*p* < 0.05).

The LFP oscillatory activities in the left and right HB recorded during rest are illustrated in Fig. 2A, C. Specifically, the oscillatory activities recorded from patient 2 were characterized by one peak at 8–10 Hz in the left HB, whereas the data from patient 3 exhibited one peak at 8–10 Hz in both the left and right HB. On the other hand, patient 4 exhibited neither a unilateral peak nor a bilateral peak in power of the oscillatory activities within the frequency ranges assessed. Similarly, patient 5 and patient 6 showed two peaks, one at 8–10 Hz and another at 15–20 Hz, in both the left and right HB, whereas patient 7 was characterized by one bilateral peak at 15–20 Hz.

#### Power-spectral density

As shown in Fig. 2B, D, moderate and large (*r* > −0.50) negative correlations existed between, on the one hand, the power of the various oscillations, except for the low-beta oscillation (13–20 Hz) and, on the other hand, the patients’ baseline HAMD and HAMA scores. This observation reflects that a lower level of oscillatory activities in the HB was associated with higher levels of depression and anxiety. The magnitude of the correlations, however, varied as a function of both the frequency band of the LFP oscillations and the HB recording side (left vs. right). We observed that the strongest negative correlation existed between the power of the high-beta oscillation (21–30 Hz) recorded from the left HB and the patients’ baseline HAMD scores (*r* = −0.928, *p* = 0.014). The most distinct correlations observed in the LFP data from the right HB were between the power of both the gamma5 oscillation (71–80 Hz) and gamma6 oscillation (81–90 Hz) and the patients’ baseline HAMA scores (*r* = −0.899, *p* = 0.019 and *r* = −0.899, *p* = 0.019, respectively).

#### Habenula asymmetry index

**Fig. 3:**
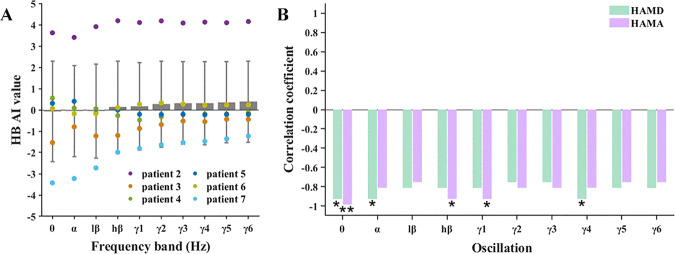
Electrophysiological results of asymmetry index. Asymmetry index (AI) of HB LFP oscillations as a function of frequency band (Panel **A**) and its correlation with baseline HAMD and HAMA scores (Panel **B**). In Panel **B**, correlations reaching statistical significance are indicated by *(*p* < 0.05) and **(*p* < 0.01).

The individual HB asymmetry values for the patients ranged from −4 to 4 (Fig. 3A). Patient 2 exhibited remarkably high positive AI values for all frequency-band oscillations (AI = 3.99 ± 0.27). This indicates that, in this patient, the oscillatory-activity level of the left HB was almost 100 times stronger than that of the right HB. In contrast, patients 3 and 7 had negative AI values for all frequency-band oscillations (AI = −0.83 ± 0.38 for patient 3, AI = −2.04 ± 0.80 for patient 7). These negative AI values indicate that the activity of their right HB was several-to-ten times stronger than that of the left HB. Patients 4, 5, and 6 had AI values close to zero, indicating that the oscillatory activities of their left and right HB were relatively balanced.

Furthermore, the AI values of all frequency-band oscillations showed strong negative correlations with the patients’ baseline HAMD and HAMA scores (Fig. 3B). These correlations indicate that a more symmetrical pattern of HB oscillatory activity was associated with higher levels of depression and anxiety. The observed correlations with the HAMD scores were the largest for the AI values of theta (*r* = −0.928, *p* = 0.014), alpha (*r* = −0.928, *p* = 0.014), and gamma4 (*r* = −0.928, *p* = 0.014) oscillations. The strongest correlations with the HAMA scores were found for the AI values of theta (*r* = −0.986, *p* = 0.001), high-beta (*r* = −0.928, *p* = 0.022), and gamma1 (*r* = −0.928, *p* = 0.022).

### Side effects

As described above, patient 2 was lost to 3-month FU because she experienced a manic episode that required hospitalization 2 months after DBS surgery. We believe that this unfortunate outcome was related to the nature of the mood disorder itself instead of the HB–DBS surgery conducted. Patient 3 reported intermittent severe back pain, which did not seem to be related to DBS. Patient 4 experienced a decline in her physical and mental status, including suicide ideation, and discontinued the treatment 2 months after surgery. Like patient 2, the outcome of patient 4 seemed to be more related to the severity and persistence of the illness than to the HB–DBS treatment. Patients 5 and 6 reported dizziness, which was transient and was eliminated by adjusting the stimulation parameters. Finally, patient 7 developed impulsive behaviors after surgery, including reckless purchases and money loaning, but these behaviors only emerged during the later phase of the study after he stopped taking his prescribed antidepressant medication.

## Discussion

In this prospective study, we explored the clinical effectiveness and safety of HB–DBS in several patients with TRD. As we hypothesized, HB–DBS treatment was associated with a relatively rapid and sustained improvement in patients’ depression symptoms, with an average symptom reduction of 49% at 1-month FU (*n* = 7) and 64% at 12-month FU (*n* = 3). The clinical response rates of partial and full responders were 86% and 100% at 1- and 12-month FU, respectively. Moreover, the patients’ comorbid anxiety symptoms showed a similar improvement pattern after surgery, with a symptom decrease of 49% at 1-month FU and 70% at 12-month FU. Additionally, the patients’ sleep quality, health status, functional impairments, and QoL all showed similar, though more variable, patterns of improvement over the 12-month treatment course. Taken together, these results support the findings from an initial treatment study [16] and indicate that HB–DBS can produce rapid and widespread clinical benefits to at least certain patients who suffer from TRD or BD.

Furthermore, the patients’ symptoms of mania were improved following HB–DBS surgery, reaching an average reduction of 59% at 1-month FU and 100% at 12-month FU. However, these improvements did not reach statistical significance. Similarly, these group-averaged data should be qualified because 1 patient developed full-blown symptoms of mania at 2-month FU that required hospitalization, while 3 other patients experienced almost no mania symptoms at baseline and throughout the study period (YMRS score of 2 or lower). Yet, two patients who presented with mild hypomania at baseline (YMRS scores of 15 and 11) did experience clinically significant improvements after HB–DBS surgery (YMRS score of 0 at 12-month FU). As discussed later, it may not be unexpected that the latter two patients were among the 3 patients who did not drop out of this pilot study.

In addition, we observed significant correlations between the patients’ LFP data recorded from the HB and the severity of depression and anxiety symptoms at baseline. In general, the presence of relatively weaker or more symmetrical LFP oscillatory activities of HB was associated with higher baseline levels of depression and anxiety. It should be noted that the latter observation appears to be inconsistent with findings of previous EEG studies, which suggested that asymmetric activity of the frontal cortex was related to depression [18, 19]. However, the present study cannot easily be compared with these studies due to differences in the brain structures of interest (HB vs. frontal cortex) and modes of electrophysiological recording used (intracerebral LFP vs. scalp EEG). Additionally, we found that the presence of a strong power-to-power cross-frequency coupling between the left and right HB oscillations was related to higher baseline levels of depression and anxiety (Supplementary Fig. 2). These correlations between the LFP and clinical data seem to support the view that HB dysfunction plays a crucial role in the pathophysiology of MDD [20]. Although HB dysfunction could be a consequence rather than a cause of mood disturbances, a recent experimental study showed that optogenetic manipulation of HB activity can readily trigger depressive-like behavior in mice [21]. Notwithstanding, the present LFP findings have not been reported before and their physiological significance, if any, remains unclear. Consequently, the findings should be regarded as preliminary and in need of confirmation before any solid conclusion can be drawn.

Substantial differences between individual patients were evident in the clinical response to HB–DBS treatment, which have frequently been observed in prior DBS treatment studies for TRD [9]. Although differences in DBS targeting and stimulation parameters are likely to contribute to individual variability in clinical response, it has been suggested that symptom severity, illness duration, and the amount and type of medication used by patients could also modify the clinical effectiveness of DBS treatment. Moreover, significant variability in treatment response may stem from the diagnosed disorder itself because neither MDD nor BD is believed to have a unique etiology and pathophysiology, representing both etiologically and biologically heterogeneous psychiatric disorders. Accordingly, it may be difficult to obtain consistent DBS treatment effects in such heterogeneous patient populations. An important goal for future research is to improve the clinical outcome of individual patients by identifying and taking into account the various sources of variability that can modify the therapeutic action of DBS treatment for mood disorders.

Finally, a possible limitation of this study is that 3 out of the 7 patients included dropped out before the study ended. Also, the 12-month FU data from another patient had to be excluded from analysis due to a failure to maintain medication adherence. Consequently, the clinical outcome data obtained at 12-month follow-up may be biased, providing an overestimation of the clinical effectiveness of HB–DBS. Indeed, when we compared the data from the three patients who were not lost to 12-month FU with the data from the four patients who were lost, the former patients displayed good clinical responses and medication adherence along with experiencing minor or no adverse side effects. There were no marked demographic or clinical differences at baseline between these patients, except for psychiatric comorbidity, which was present among the latter patients but absent among the former patients. Thus, the 12-month FU data seem to provide a somewhat biased, inflated view on the clinical effectiveness of HB–DBS. Yet, it should be underlined that substantial clinical benefits were observed already at 1-month FU, when none of the patients had dropped out, and that these benefits were generally maintained throughout the study. These results are preliminary but promising and warrant larger and well-controlled clinical studies into the safety and efficacy of HB–DBS for treatment-refractory mood disorders.

## Supplementary information

Supplementary Table 1

Supplementary Figure 1

Supplementary Figure 2

supplementary legends
