# Supplementary material for: Bilateral Habenula deep brain stimulation for treatment-resistant depression: clinical findings and electrophysiological features
Source: Transl Psychiatry. 2022 Feb 3;12:52. doi: 10.1038/s41398-022-01818-z (PMC8813927; doi:10.1038/s41398-022-01818-z)
Supplement: Supplementary file 1 — Supplementary Table 1 [file 41398_2022_1818_MOESM1_ESM.docx]

Supplementary Table 1. Deep brain stimulation parameters for each patient

|  | **Left** | | | | **Right** | | | |
| --- | --- | --- | --- | --- | --- | --- | --- | --- |
|  | **Contact** | **Voltage** | **Pulse** | **Frequency** | **Contact** | **Voltage** | **Pulse** | **Frequency** |
| **Patient 1** | C+0- | 2 | 60 | 60 | C+1- | 2 | 60 | 60 |
| **Patient 2** | C+1- | 2 | 60 | 130 | C+1- | 2 | 60 | 130 |
| **Patient 3** | 2+1- | 1.6 | 60 | 60 | 2+1- | 1.6 | 60 | 60 |
| **Patient 4** | C+0-1- | 2.25 | 50 | 130 | C+1- | 3.45 | 120 | 160 |
| **Patient 5** | C+1-2- | 1.7 | 60 | 130 | C+1-2- | 1.7 | 60 | 130 |
| **Patient 6** | 3+2- | 3.5 | 60 | 130 | 3+2- | 3.5 | 60 | 130 |
| **Patient 7** | C+1- | 3 | 90 | 160 | C+0- | 2.9 | 90 | 160 |

0 1 2 3: from ventral to dorsal.
