# Supplementary figures and images for: Bilateral Habenula deep brain stimulation for treatment-resistant depression: clinical findings and electrophysiological features
Source: Transl Psychiatry. 2022 Feb 3;12:52. doi: 10.1038/s41398-022-01818-z (PMC8813927; doi:10.1038/s41398-022-01818-z)

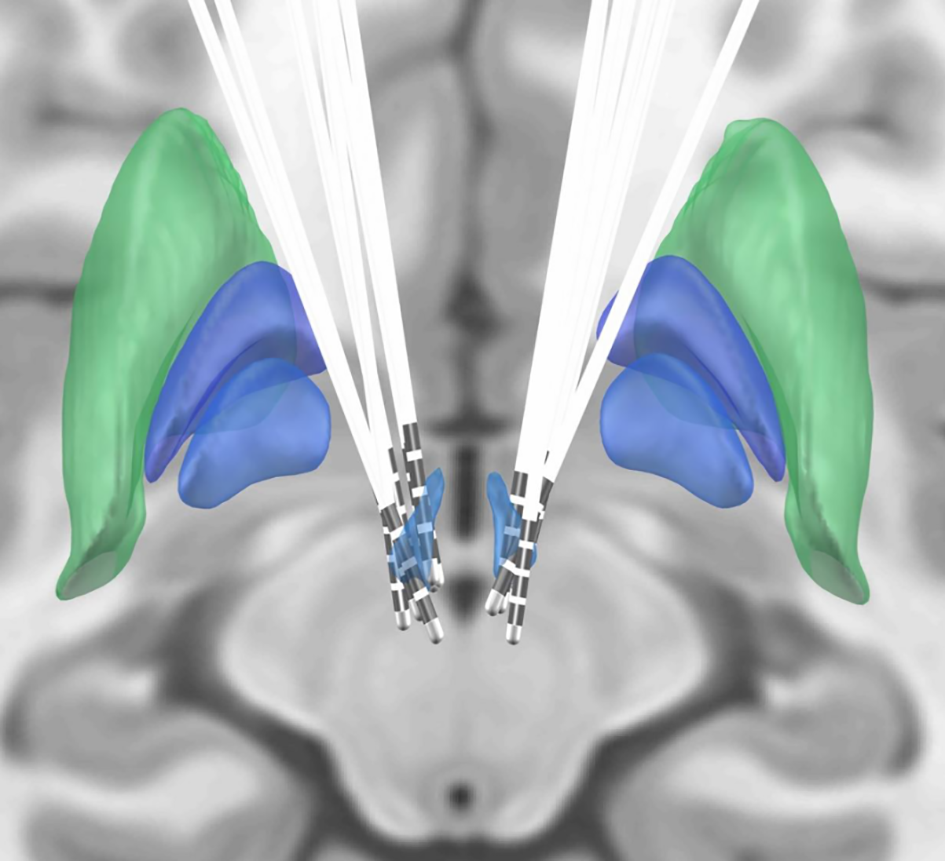

Supplement: Supplementary file 2 — Supplementary Figure 1 [file 41398_2022_1818_MOESM2_ESM.tif]
