# Supplementary material for: Bilateral Habenula deep brain stimulation for treatment-resistant depression: clinical findings and electrophysiological features
Source: Transl Psychiatry. 2022 Feb 3;12:52. doi: 10.1038/s41398-022-01818-z (PMC8813927; doi:10.1038/s41398-022-01818-z)
Supplement: Supplementary file 4 — supplementary legends [file 41398_2022_1818_MOESM4_ESM.docx]

**Supplementary Figure 1. DBS electrode location reconstruction.** Pu: putamen, Gpe: globus pallidus externus, GPi: globus pallidus internus, HB: habenula.

**Supplementary Figure 2.** Average cross-frequency correlation (CFC) between left and right HB oscillations (Panel A) and its correlation with baseline HAMD (Panel B) and HAMA (Panel C) scores. In Panels B and C, correlations reaching statistical significance are indicated by * (p < 0.05) and ** (p < 0.01).

**Supplementary Table 1.** DBS parameters at final follow-up for each patient
